# Macrophage phenotype and its relationship with renal function in human diabetic nephropathy

**DOI:** 10.1371/journal.pone.0221991

**Published:** 2019-09-11

**Authors:** Xiaoliang Zhang, Ying Yang, Yu Zhao

**Affiliations:** Institute of Nephrology, Zhong Da Hospital, Southeast University, School of Medicine, Nanjing, Jiangsu, China; Baker IDI Heart and Diabetes Institute, AUSTRALIA

## Abstract

This study aimed to examine the macrophage phenotype and its relationship to renal function and histological changes in human DN and the effect of TREM-1 on high-glucose-induced macrophage activation. We observed that in renal tissue biopsies, the expression of CD68 and M1 was apparent in the glomeruli and interstitium, while accumulation of M2 and TREM-1 was primarily observed in the interstitium. The numbers of CD68, M1, and M2 macrophages infiltrating in the DN group were increased in a process-dependent manner compared with the control group, and the intensities of the infiltrates were proportional to the rate of subsequent decline in renal function. M1 macrophages were recruited into the kidney at an early stage (I+IIa) of DN. The M1-to-M2 macrophage ratio peaked at this time, whereas M2 macrophages predominated at later time points (III) when the percentage of M1/M2 macrophages was at its lowest level. In an in vitro study, we showed that under high glucose conditions, macrophages began to up-regulate their expression of TREM-1, M1, and marker iNOS and decreased the M2 marker MR. However, the above effects of high-glucose were abolished when TREM-1 expression was inhibited by TREM-1 siRNA. In conclusion, our study demonstrated that there was a positive correlation between the M1/M2 activation state and the progress of DN, and TREM-1 played an important role in high-glucose-induced macrophage phenotype transformation.

## Introduction

Diabetes mellitus (DM) is one of the most common chronic diseases and, increasingly, a major cause of morbidity and mortality worldwide. DM with diabetic complications is becoming a highly important public health issue. Diabetic nephropathy (DN) is the most severe renal complication of DM, and it remains the largest single cause of end-stage renal disease (ESRD) [1–3]. Although DN is traditionally considered a nonimmune disease, accumulating evidence now indicates that immunological and inflammatory mechanisms play a significant role in its development and progression [3].

In experimental and human DN, macrophages are key inflammatory cells medicating renal injury through a variety of mechanisms, including production of reactive oxygen species, cytokines and proteases [4]. Previously, the degree of macrophage accumulation was thought to correlate with the severity of renal injury and be predictive of disease progression [5]. However, macrophages are heterogeneous and plastic cells. In response to cytokine cues, these cells undergo differentiation into two distinct subsets that are categorized as either classically activated (M1) or alternatively activated (M2). M1 (induced by IFN-γ or LPS) are associated with high microbicidal activity, proinflammatory cytokine production and tissue injury, while M2 (stimulated by IL-4 or IL-13) releases trophic cytokines, down-regulates inflammation and promotes wound healing [6–8]. More importantly, macrophages do not remain committed to a single activation [9]. M1–M2 polarization of macrophages is a highly dynamic process, and the phenotype of polarized macrophages can be reversed under physiological and pathological conditions [6]. This finding emphasizes the need for further research investigating macrophage function and phenotype at different time points during the course of the kidney disease [10].

Triggering receptor expressed on myeloid cells (TREM) is a newly identified activating receptor of the immunoglobulin superfamily present on human myeloid cells [11]. TREM-1, the first member of the TREM family to be identified, is selectively expressed on neutrophils, monocytes and macrophages and implicated in the amplification of inflammatory responses by coordinating with the signal pathway mediated by Toll-like and NOD-like receptors [12, 13]. Recent studies showed that in obstructive nephropathy, TREM-1 can modulate macrophage polarization by inhibiting M1 macrophage activation and enhancing M2 macrophage activation, and plays a pivotal role in the development of the disease [14].

Therefore, the aim of the current study was to examine macrophage function and phenotype at different pathological stages during the process of DN and under high glucose conditions to investigate the role of TREM-1 on the macrophage activation state.

## Materials and methods

### Patients and pathological classification

IEC for Clinical Research of Zhongda Hospital, Affilited to Southeast University (2015ZDKYSB002) approved this study. We retrospectively studied 46 patients with DN who were confirmed by diagnosis of a renal biopsy between 2011 and 2015 at Southeast University School of Medicine Affiliated Zhong Da Hospital. The other four normal renal tissue specimens, taken from patients with renal trauma or renal tumors, were the control group. Pathologic classification of diabetic nephropathy was referred to Thijs W' article which pubilshed on JASN in 2010 [15].

### Renal pathology

Kidney tissue from the DN and control were fixed in 10% formalin solution and embedded in paraffin. Sections (2 μm) were stained with the periodic acid-Schiff reagent and counterstained with hematoxylin. Digital images of glomeruli and interstitium areas were obtained from light microscopy (magnification ×200).

### Immunohistochemistry

Immunohistochemistry was performed on formalin-fixed, paraffin-embedded sections (3 μm) using a microwave based antigen retrieval technique. Sections were treated with 0.3% hydrogen peroxide to quench the endogenous peroxidase activity. Titrated primary antibodies against the following antigens were used: for humans, anti-CD68 (Novues, USA), anti-MR (R&D Systems, USA), anti-TREM-1 (Sigma, USA). Next, the samples were incubated with the appropriate secondary antibodies. The immunostaining was visualized using the diaminobenzidine substrate system, and the slides were counterstained with hematoxylin. CD68^+^, MR^+^ and TREM-1^+^ glomerular and interstitial infiltrating cells in the cortex were counted blindly in at least 5 high-power (magnification ×400) fields. The number of M1 macrophages was equal to the number of CD68+ macrophages minus the number of MR+ macrophages. Data were converted to cells/gcs and cells %/area.

### Immunofluorescence

Antigens were retrieved by microwaving paraffin-fixed sections (3 μm). CD68, MR and iNOS antibodies from Abcam were detected using goat anti-rabbit and goat anti-mouse secondary antibodies (Jackson, America). After staining the nuclei with DAPI, double-immunostaining for CD68 and iNOS, CD68 and MR were visualized with a fluorescence microscope (magnification ×400).

### Cell culture

Murine macrophage cells (RAW264.7), purchased from Shanghai Bogoo Biotechnology Company (Shanghai, China), were routinely maintained in RPMI 1640 media (containing 11.1 mM glucose) supplemented with 10% fetal bovine serum (Sciencell, USA) and incubated at 37°C in 5% CO2. Firstly, RAW264.7 cells were stimulated with 25 mM high glucose for 24 h. Second, in order to examine the effect of TREM-1 on high-glucose induced macrophage polarization, the RAW264.7 cells were treated with TREM-1 siRNA (Invitrogen, America) and the cells were washed three times with PBS followed by RNA harvest for quantitative real-time polymerase chain reactions (RT-PCR) and the proteins for western blotting.

### Treatment of cells with siRNA

TREM-1 siRNA and disorderly NC sequences were designed and synthesized and the TREM-1 siRNA sequences were as follows: TREM-1 siRNA-1 (sense: 5'-CCUGGUCUUGGAGUCACUAUCAUAA-3', antisense: 5'-UUAUGAUAGUGACUCCAAGACCAGG-3'), TREM-1 siRNA-2 (sense: 5'-UCVVGUGACAGACUCUGGAUUGUAU-3', antisense: 5'-AUACAAUCCAGAGUCUGUCACUUGA-3'), TREM-1 siRNA-3 (sense: 5'-CAUUGUUCUAGAGGAAGAAAGGUAU-3', antisense: 5'-AUACCUUUCUUCCUCUAGAACAAUG-3'). RAW264.7 macrophages were transfected with either non-specific siRNA oligomers or Stealth siRNAs targeting TREM-1 mRNA by using the RNAiMAX reagent according to the manufacturer's instructions. Before transfection, the cells were seeded in 6-well plates at 1×105 cells/well and incubated in RPMI 1640 containing 10% FBS for 24 h. After the cells achieved 50% to 70% confluence, they were washed twice with PBS before adding fresh medium, and siRNA⁃lipid complexes containing TREM-1 siRNA were formed by incubating 50 pmol of each siRNA duplex with 7.5 μl of RNAiMAX for 20 min at room temperature in a total volume of 250 μl of RPMI without antibiotics. The liposomes were added to the cells, and siRNA treatment was continued for 24 h. Silencing of TREM-1 at the gene and protein level was verified by RT-PCR and western blotting.

### Quantitative RT-PCR

TRIzol (TaKaRa, Japan) was used to isolate total cellular RNA from RAW264.7 cells according to the manufacturer's protocol. According to the manufacturer’s recommendations, cDNA synthesis was performed using the High-Capacity cDNA Reverse Transcription Kit (Thermo Fisher Scientific, China) and SuperScript III Reverse Transcription (Thermo Fisher Scientific, China). For the RT-qPCR, the SyBR Select Master Mix and the ViiA 7 instrument was been used from Thermo Fisher Scientific. All of the PCR primers were synthesized by Shanghai Generay Biotechnology Company (Shanghai, China). The primer sequences were as follows: Mouse iNOS (5'-TCTTGGAGCGAGTTGTGGATGT-3' forward; 5'-TAGGTGAGGGCTTGGCTGAGTG-3' reverse), mouse MR (5'-CCTCAGCAAGCGATGTGCCTAC-3' forward; 5'-GTCCCCACCCTCCTTCCTACAA-3' reverse), mouse TREM-1(5'- GACTGCTGTGCGTGTTCTTTG -3' forward; 5'- GCCAAGCCTTCTGGCTGTT -3' reverse), and β-actin (5'-CCCAAAGCTAACCGGGAGAAG-3' forward; 5'-GACAGCACCGCCTGGATAG-3' reverse). Real-time PCR was performed on an ABI PRISM 7300 real-time PCR System (Applied Biosystems, USA). The reaction conditions were as follows: melting for 15 minutes at 37°C, 5 seconds at 95°C and 40 cycles of two-step PCR including melting for 5 seconds at 95°C and annealing for 31 seconds at 60°C. The 2-△△Ct method was used to determine the relative amounts of product using β-actin as an endogenous control.

### Western blot analysis

Total protein was extracted from the RAW264.7 cells using a Total Cell Protein Extraction Kit (Kaiji, Nanjing, China) according to the to the manufacturer’s instructions. Proteins (70 μg) were separated by sodium dodecyl sulfate polyacrylamide gel electrophoresis (SDS-PAGE) and transferred to a nitrocellulose membrane. The membranes were then incubated overnight at 4°C with the primary antibodies against iNOS, MR, TREM-1 and β-actin. After three washes with PBST/5 min, horseradish peroxidase-conjugated secondary antibody at a 1:5000 dilution was added to incubate with the nitrocellulose membrane for 1–2 hours. Finally, the membranes were visualized with an enhanced chemiluminescence advanced system (GE Healthcare, UK) and captured on X-ray film. Immunoreactive bands were quantified with densitometry using ImageJ software (NIH, USA).

### Flow cytometry

RAW264.7 cells obtained after different intervention conditions and characterized by fluorescence activated cell sorting (FACS) analysis after immunostaining with monoclonal antibodies against the M1 markers APC-CD11b (0.20μg per million cells in 100μl volume, Biolegend, USA) or M2 markers APC-CD206 (0.20μg per million cells in 100μl volume, Biolegend, USA), FITC- CD68 (0.20μg per million cells in 100μl volume, Biolegend, USA).

### Statistical analysis

Results are expressed as the mean ± standard deviation (SD). Statistical analysis was assessed using one way analysis of variance followed by the least-significant difference test or Tamhane's test and was analyzed with SPSS 16.0. Associations between parameters were examined by calculating Spearman’s correlation coefficient. A p-value <0.05 was considered to be significant.

## Results

### Changes of renal histopathology, macrophage phenotype and TREM-1 expression in human DN

**Fig 1:**
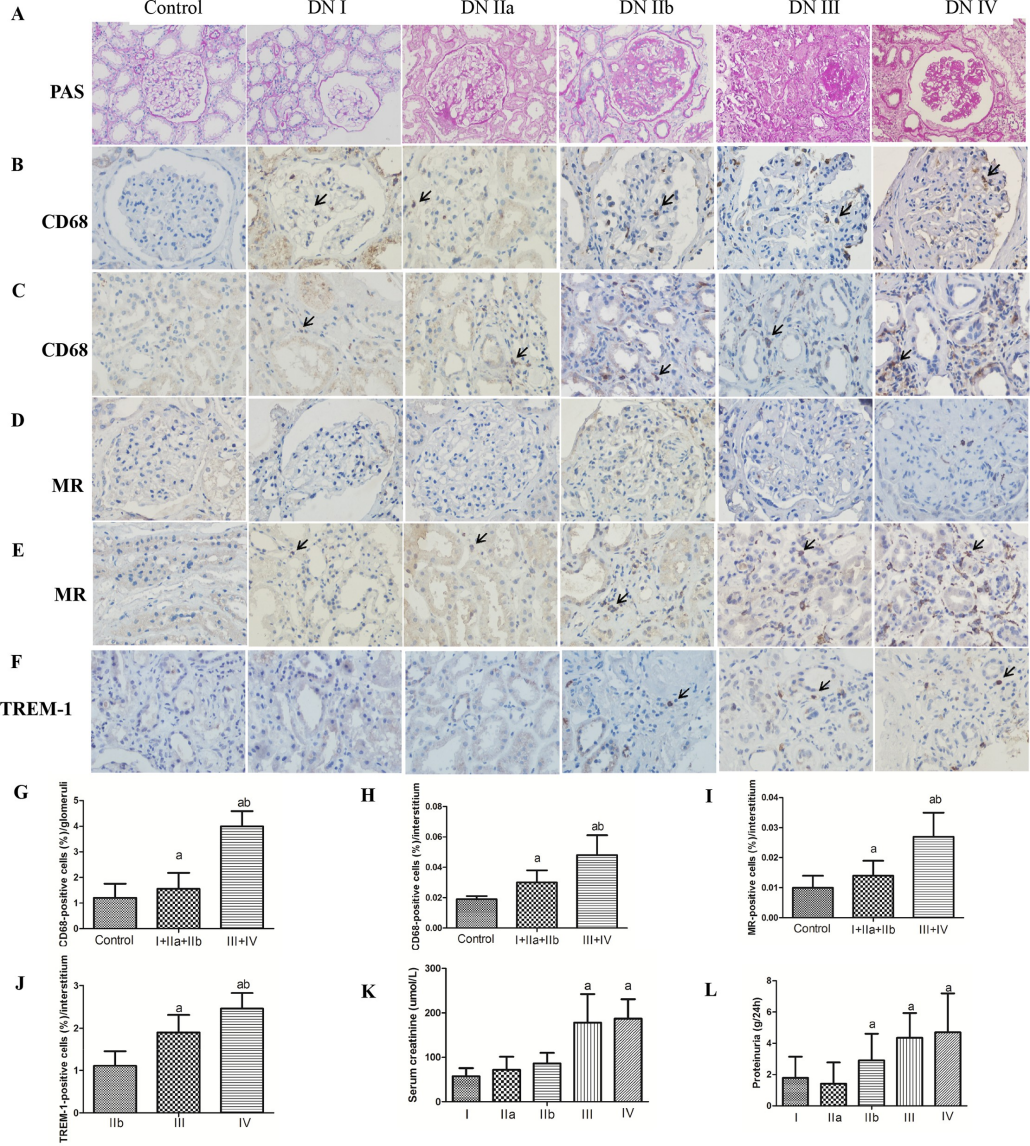
Histopathological features and the expression of CD68, MR, and TREM-1 in the kidneys of diabetic nephropathy patients. A: PAS stains of patients' renal tissues (×200, bar = 100μm). B–E: Immunohistochemical staining of CD68, MR and TREM-1 (black arrows) in glomeruli and the interstitial areas (×400, bar = 50μm).). F–H: Quantification of the number of CD68, MR TREM-1. The data are presented as the mean±SD (n = 6 per group). ap<0.05 vs. control, bp<0.05 vs. I+IIa+IIb.

Human DN displayed a severe kidney morphological injury compared with the control group and was characterized by glomerular hypertrophy, thickening of the glomerular basement membrane and accumulation of extracellular matrix that finally resulted in tubulointerstitial and glomerular fibrosis and Kimmelstiel–Wilson lesions (Fig 1). As the disease progresses, serum creatinine and proteinuria gradually increase (Fig 1K and 1L).

**Fig 2:**
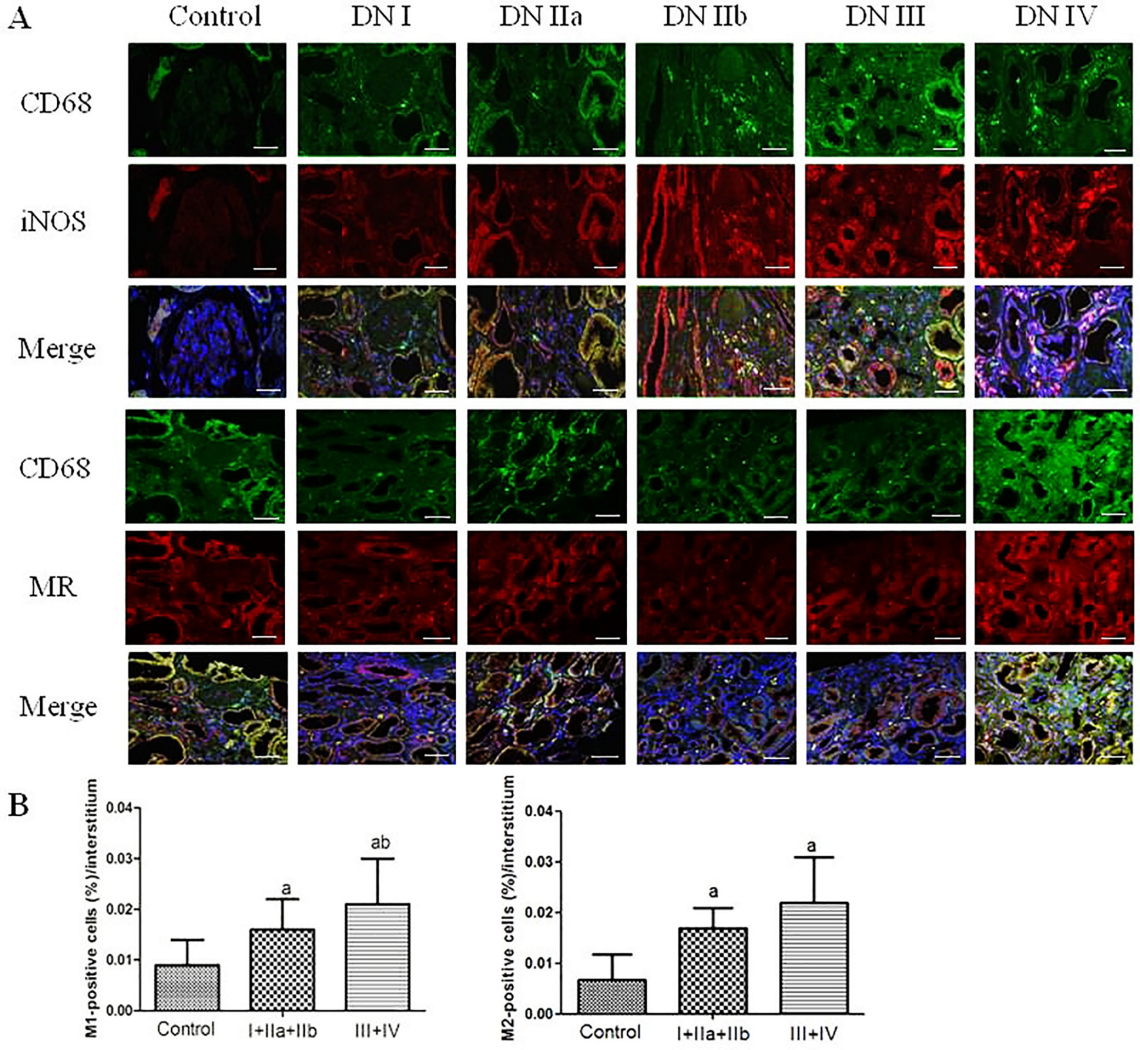
Identification of M1 macrophages in diabetic glomeruli and interstitium. A: Immunofluorescent staining for M1 macrophages (iNOS+CD68) and M2 macrophages (MR+CD68) in control and DN patients(×400, bar = 50μm).). The data are presented as the mean±SD (n = 6 per group). ap<0.05 vs. control, bp<0.05 vs. I+IIa+IIb.

In biopsies of renal tissue with human diabetic nephropathy, with the progress of DN, CD68, M1 macrophages (iNOS), M2 macrophages (MR) and TREM-1 were mainly detected in interstitium and significantly increased compared with the control group. Moreover, the expression of CD68, M1, M2, TREM-1 were significantly higher in DN late stage (III + IV) than in DN early stage (I + IIa + IIb) (Figs 1 and 2).

### Relationship between macrophage phenotype, TREM-1 and renal function

**Fig 3:**
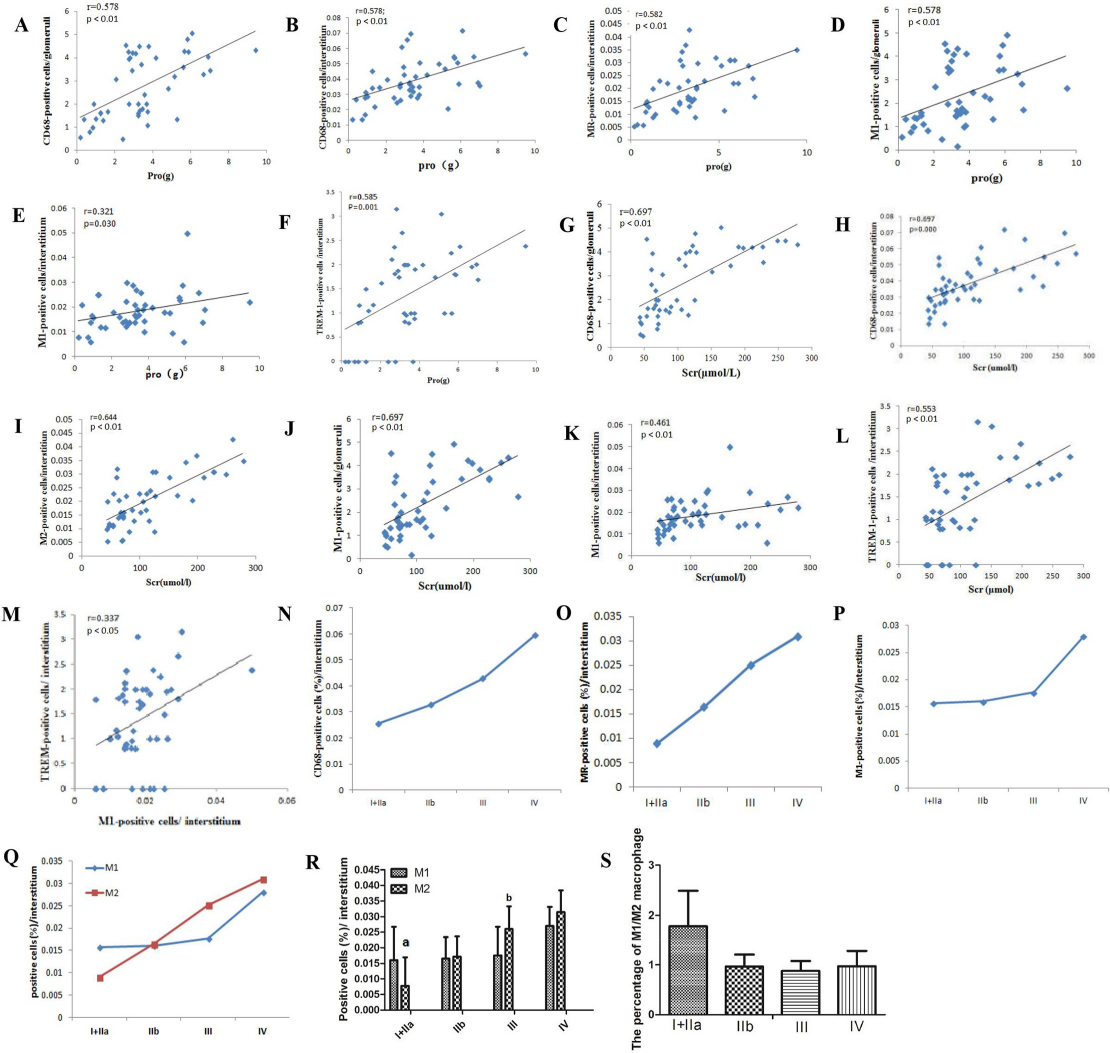
Correlations between CD68, TREM-1, M1/M2 macrophages and serum creatinine or proteinuria. A–L: The correlation between different markers and renal function. r = Spearman’s correlation coefficient. N–R: Correlations between M1/M2 activation state and the progress of DN. S: The percentage of M1/M2 macrophages at different stages of DN. The data are presented as the mean±SD (n = 6 per group). ap<0.05 vs. M1(I+IIa), bp<0.05 vs.M1(III).

As shown in Fig 3, glomerular CD68, iNOS (an M1 macrophage marker) correlated with proteinuria (r = 0.578, p < 0.001; r = 0.578, p < 0.001) and serum creatinine (r = 0.697, p < 0.001; r = 0.697, p < 0.001). Likewise, there were positive correlations between interstitial CD68, iNOS (an M1 macrophage marker), MR (an M2 macrophage marker), TREM-1, proteinuria (r = 0.578, p < 0.001; r = 0.321, p = 0.030; r = 0.582, p < 0.001; r = 0.585, p < 0.001) and serum creatinine(r = 0.697, p < 0.001; r = 0.461, p = 0.001; r = 0.644, p < 0.001; r = 0.553, p < 0.001).

### Macrophage activation state during the different pathological phases of DN

As shown in Fig 3, positive correlations were observed between the protein expression of TREM-1 and M1 (r = 0.337, p = 0.022). M1/M2 macrophage infiltration strongly correlated with the progress of DN. An interesting feature of the early phase (I+IIa) of DN was an increase in M1 macrophage accumulation, and the percentage of M1/M2 macrophages reached its maximum. However, during the late period (III) of DN, M2 macrophage was increased, and the ratio of M1 and M2 macrophage was at its lowest level.

### High glucose induces macrophages towards a M1 phenotype and increases the expression of TREM-1 in vitro

**Fig 4:**
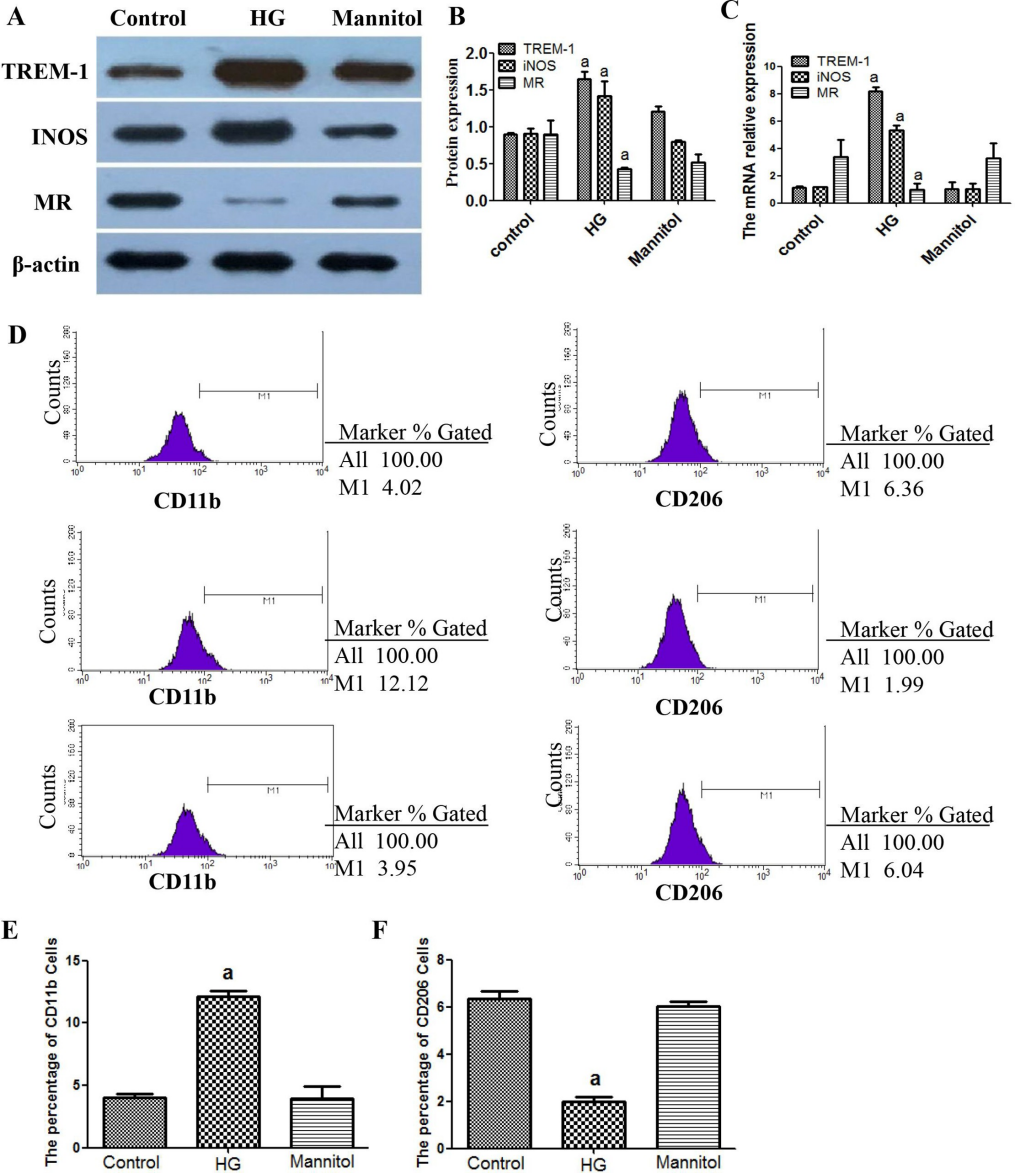
The effect of high glucose on M1/M2 macrophage-specific markers and TREM-1 expression. RAW264.7 cells were treated with 25mM glucose (HG) for 24h. The cells were collected for western blot (A and B) and RT-PCR (C) and flow cytometry (D, E and F) analysis. β-actin was used as an internal control. A concentration of 11.1mM glucose was used as the control. Results are the mean±SD (n = 3). ap < 0.05 vs control.

To investigate the effect of high glucose on the macrophage phenotype, RAW264.7 cells were stimulated with 25 mM high glucose for 24 h, and the iNOS (an M1 marker), MR (an M2 marker) and TREM-1 were measured. iNOS and TREM-1 expression were up-regulated, while MR was down-regulated by high glucose compared with the control group. No significant differences in the levels of iNOS, MR or TREM-1 were found between the mannitol and the control group, which excluded the influence of hyperosmolarity (Fig 4).

### TREM-1 siRNA inhibits M1 macrophage activation and enhances M2 macrophage activation in vitro

**Fig 5:**
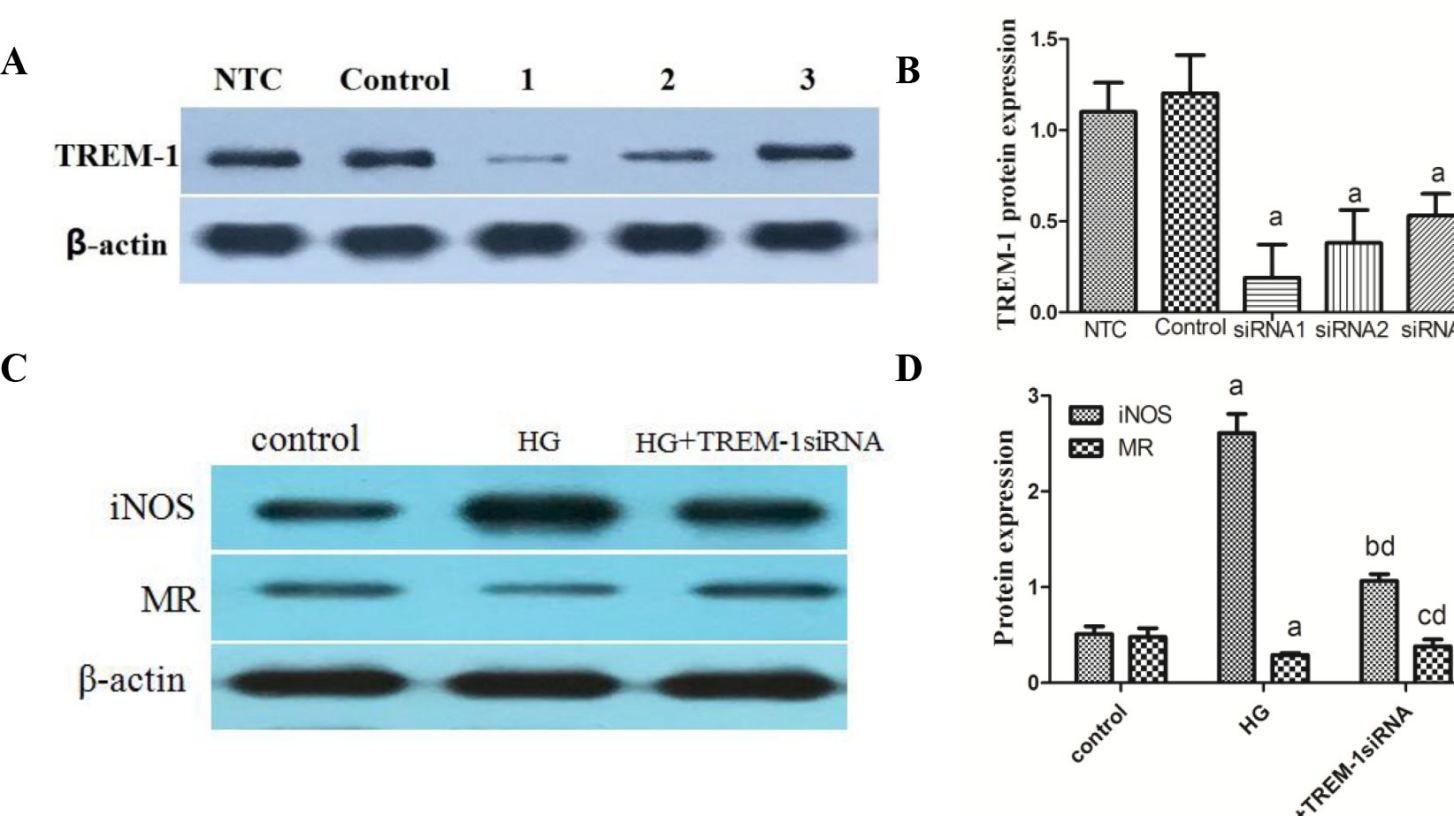
Effects of TREM-1 siRNA on M1/M2 macrophage-specific markers expression. RAW264.7 cells were treated with 25 mM glucose (HG) for 24 h. β-actin was used as an internal control. A concentration of 11.1 mM glucose was used as the control. The data are presented as the mean±S (n = 3). ap<0.05 vs. control, bp<0.05 vs. HG.

To determine the role of TREM-1 in the high-glucose-induced macrophage activation state, TREM-1-siRNA was administered to the RAW264.7 cells to inhibit TREM-1 and a non-target control (NTC) siRNA was used to eliminate the non-specific effects of the transfection reagents. Compared with the control group, the levels of transcription and protein significantly decreased in all three TREM-1 siRNA groups, whereas no significant differences were observed between the control and NTC siRNA group. The inhibition ratios of TREM-1-siRNA-1, 2 and 3 were 89.2%, 70.4% and 63.7%, respectively. Therefore, we used TREM-1-siRNA-1 as the best intervention siRNA (Fig 5A and 5B).

As shown in Fig 5, TREM-1 knockdown significantly inhibited the high glucose induced increase in iNOS mRNA (high-glucose versus TREM-1-siRNA: 5.048 ± 0.645 versus 2.260 ± 0.062) and decrease in MR mRNA expression (high-glucose versus TREM-1-siRNA: 1.042 ± 0.036 versus 2.214 ± 0.083). The data indicated that inhibition of TREM-1 expression eliminated the high glucose-induced macrophage phenotype switch to M1. The changes in the protein expression levels were in keeping with those of the mRNA.

## Discussion

DM is one of the main risk factors for developing chronic kidney disease. The risk of developing nephropathy is approximately 30% and 20% in DM1 and DM2, respectively. DN is the most common cause of ESRD, and both the incidence and prevalence of DN continue to increase [2, 3, 16]. The molecular mechanisms responsible for its development are complex and not completely understood [16, 17]. The classic view considered metabolic and hemodynamic alterations as the main causes lead to renal injury in diabetes [3]. However, recent studies have shown that inflammation-related molecules and pathways are critically involved in the pathophysiology of DN. while a substantial increase in tissue macrophages is a common feature of kidney disease and play an important role in the process [18, 19]. Chow et al. showed macrophages account for almost all kidney leucocyte infiltration in this disease and their accumulation is associated with both the progression of diabetes (hyperglycemia, glycosylated hemoglobin) and the severity of kidney damage (histological lesions, renal dysfunction) in Type 2 diabetic db/db mice [4, 20].

Our study results suggest that CD68, M1 and M2 macrophages infiltrated into the glomeruli and interstitium, even in the early stage of DN with nonspecific histological renal changes. The number of infiltrations was higher than that in the control group, increased progressively with the duration of diabetes and was correlated with serum creatinine and proteinuria. These results are consistent with both human and experimental studies, and establish the importance of macrophages in the progression of DN. A body of evidence supports the hypothesis that macrophages can induce renal injury through interacting with resident renal cells or be activated by components of the diabetic milieu, which lead to the production of a host of proinflammatory and profibrotic factors [4, 21, 22], but direct proof of the role and mechanism of macrophages in the entire process of DN has been lacking.

Macrophages are heterogeneous and plastic cells, and adapt to their surrounding microenvironment by undergoing two different polarization states: the classically activated M1 phenotype and the alternatively activated M2 phenotype [23, 24]. The M1 phenotype is characterized by high production of reactive nitrogen and oxygen intermediates and plays a central role in inflammation and host defense. In contrast, M2 macrophages are considered to have immunoregulatory functions and to be involved in tissue remodeling, repair and healing [8, 24]. The identification of M1 and M2 macrophages relies on a combination of membrane receptors, cytokines, chemokines, and effector mediators [25].

Wang et al. demonstrated that in severe combined immunodeficient (SCID) mice with adriamycin nephropathy, injection of M1 macrophages stimulated with LPS worsened their histological and functional injury, whereas administration of IL-4 and IL-13 activated M2 macrophages reduced the severity of renal damage and promoted repair [26, 27]. Furthermore, Lee et al. observed an increase in the numbers of iNOS-positive pro-inflammatory (M1) macrophages in the first 48 hours after ischemia/reperfusion injury, whereas arginase 1- and mannose receptor-positive non-inflammatory (M2) macrophages predominated during the recovery stage, indicating that M2 plays an important role in injury repair [28]. These results are consistent with the suggestion of Han et al. in that they found macrophage infiltration decreased with an apparent change from a pro-inflammatory M1 phenotype to an alternatively activated M2 phenotype during the fibrotic phase of rat crescentic glomerulonephritis [29]. Our previous study also found that streptozocin (STZ)-induced DN rats show increased M1 macrophages in the early stage of the disease, followed by progression of histopathological lesions and renal dysfunction, while M2 macrophages inhibited inflammation and attenuated podocyte impairment and facilitated wound healing [30, 31]. All of the above studies reveal different diseases and changes in the renal microenvironments determine macrophage activation states, while the development and prognosis of kidney diseases are finally dominated by macrophage phenotypes [32].

Our study showed the initial influx of macrophage is the M1 phenotype in the early stage (I+IIa) of DN, and the ratio of M1 and M2 macrophages is at its highest level. However, in response to progression of the disease, there is a subsequent switch to an alternatively activated macrophage phenotype, and most of the interstitial macrophage infiltration is the M2 phenotype during the late stage (III) of DN when the percentage of M1/M2 falls to its lowest level. This finding indicates that as DN progresses; macrophages undergo a phenotype shift with a change from a classically activated M1 to an alternatively activated M2. This phenotypic switch reflects a critical role for different macrophage states in the different pathological stages of DN.

Current clinical therapies for diabetic nephropathy target regulation of the development of hyperglycemia, hyperlipidemia, and hypertension, but a large number of DM patients ultimately progress to DN [33]. As a result, a search for techniques for modulating macrophage activation based on the macrophage functional diversity is of great importance [34]. TREM-1 is a recently discovered cell surface receptor of the immunoglobulin superfamily member selectively expressed on neutrophils and subsets of monocytes and tissue macrophages [35, 36]. Human TREM-1 is a 30 kDa glycoprotein [37]. This protein consists of a single extracellular immunoglobulin-like domain of the V-type, a transmembrane region, and a short cytoplasmic tail, and associates with DAP12 for signaling and function [38]. In response to receptor ligation, activation of TREM-1/DAP12 signaling is implicated in the amplification of inflammatory responses by potentiating the secretion of proinflammatory chemokines and cytokines [13]. A recent study in a mouse model of experimental unilateral ureteral obstruction found loss of TREM-1 attenuated activation of M1 macrophages, resulting in reduced renal pathology [14]. In addition, TREM-1 deficiency attenuated Kupffer cell activation by down-regulating cytokine production and signal induction and controlled the development of hepatocellular carcinoma [39].

In an in vivo study, we reported that TREM-1 expression in renal interstitium is significantly correlated with the DN progression. In an in vitro study, under high glucose conditions, RAW264.7 cells exhibited an M1 phenotype, expressing high iNOS and up-regulated TREM-1 but with inhibition of M2 marker MR. Intriguingly, after TREM-1 siRNA treatment, the M1 marker iNOS was decreased, while the M2 marker MR was increased, indicating that the absence of TREM-1 induced a switch in high glucose-induced macrophages from the M1 to the M2 phenotype. Our data demonstrated that TREM-1 critically modulates macrophage polarization.

## Conclusions

This study established that the number of macrophages was significantly increased, and the intensity of the infiltration correlated strongly with the classes of DN, and for the first time, we demonstrated the M1/M2 activation state correlated strongly with the progress of DN, while TREM-1 played a critical role in the high-glucose induced macrophage phenotype switch. Taken together, these findings help to elucidate the different effects of macrophage phenotypes in diabetic nephropathy.

## Supporting information

S1 Fig Identification of CD68, M1, and M2 macrophages in diabetic glomeruli and interstitium. Glomerular (A) and interstitial (B) CD68-positive macrophages accumulation in control and DN at I+IIa+IIb, III+IV. Interstitial MR-positive macrophages (C) and M1 macrophages accumulation (D) in control and DN at I+IIa+IIb, III+IV. Results are the means±SE. ^a^p<0.05 vs. control, ^b^p<0.05 vs. I+IIa+IIb. ^a^p<0.05 vs. control, ^b^p<0.05 vs. I+IIa+IIb. Interstitial TREM-1-positive cells accumulation in control and IIb, III and IV of DN (E). M1 macrophages amount equals the number of CD68^+^ macrophages minus MR^+^ macrophages. ^a^p<0.05 vs.IIb, ^b^p<0.05 vs. III.
(DOC)

S1 Table Clinical parameters of DN patients (n = 46). (DOC)

S2 Table The mRNA expression of iNOS, TREM-1 and MR of each group. (RT-PCR). Control: 11.1mM glucose; HG: 25mM glucose; Results are the mean ± SD (n = 3–4 per group). aP<0.05 vs control.
(DOC)
