# Supplementary material for: Macrophage phenotype and its relationship with renal function in human diabetic nephropathy
Source: PLoS One. 2019 Sep 11;14(9):e0221991. doi: 10.1371/journal.pone.0221991 (PMC6738594; doi:10.1371/journal.pone.0221991)
Supplement: S1 Fig — Glomerular (A) and interstitial (B) CD68-positive macrophages accumulation in control and DN at I+IIa+IIb, III+IV. Interstitial MR-positive macrophages (C) and M1 macrophages accumulation (D) in control and DN at I+IIa+IIb, III+IV. Results are the means±SE. ap<0.05 vs. control, bp<0.05 vs. I+IIa+IIb. ap<0.05 vs. control, bp<0.05 vs. I+IIa+IIb. Interstitial TREM-1-positive cells accumulation in control and IIb, III and IV of DN (E). M1 macrophages amount equals the number of CD68+ macrophages minus MR+ macrophages. ap<0.05 vs.IIb, bp<0.05 vs. III. (DOC) [file pone.0221991.s001.doc]

Supporting Information


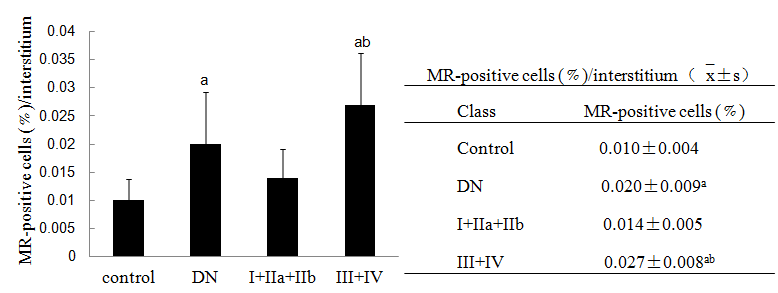

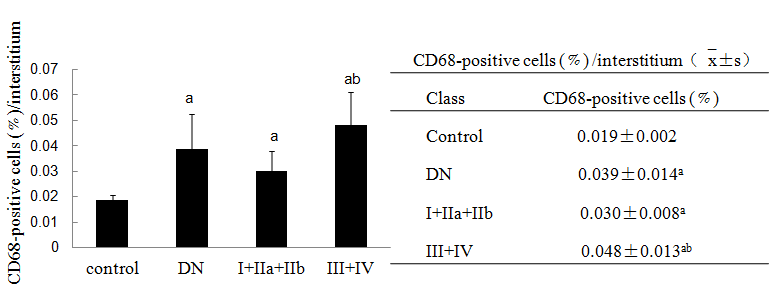

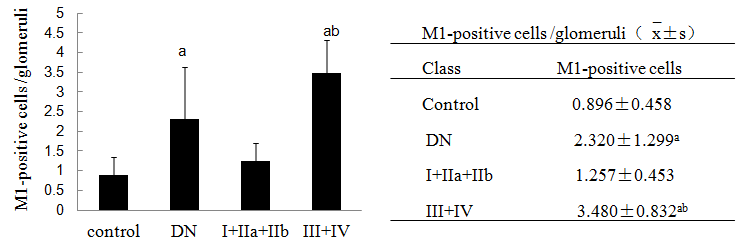

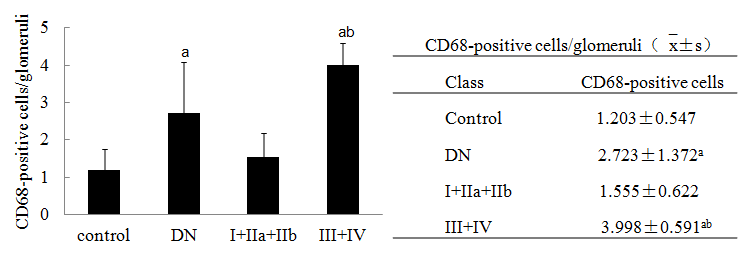

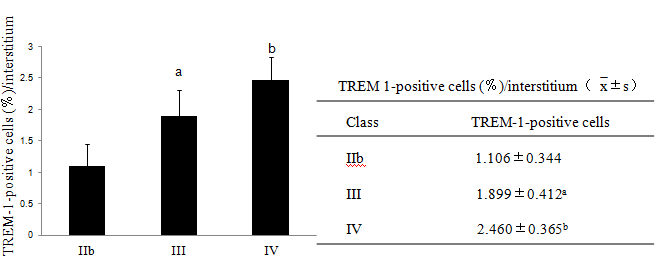


A

B

C

D

E

S1 Fig. Identification of CD68, M1, and M2 macrophages in diabetic glomeruli and interstitium. Glomerular (A) and interstitial (B) CD68-positive macrophages accumulation in control and DN at I+IIa+IIb, III+IV. Interstitial MR-positive macrophages (C) and M1 macrophages accumulation (D) in control and DN at I+IIa+IIb, III+IV. Results are the means±SE. ^a^p<0.05 vs. control, ^b^p<0.05 vs. I+IIa+IIb. ^a^p<0.05 vs. control, ^b^p<0.05 vs. I+IIa+IIb. Interstitial TREM-1-positive cells accumulation in control and IIb, III and IV of DN (E). M1 macrophages amount equals the number of CD68^+^ macrophages minus MR^+^ macrophages. ^a^p<0.05 vs.IIb , ^b^p<0.05 vs. III.
