# Supplementary material for: Macrophage phenotype and its relationship with renal function in human diabetic nephropathy
Source: PLoS One. 2019 Sep 11;14(9):e0221991. doi: 10.1371/journal.pone.0221991 (PMC6738594; doi:10.1371/journal.pone.0221991)
Supplement: S1 Table — (DOC) [file pone.0221991.s002.doc]

Supporting Information

S1 Table. Clinical parameters of DN patients (n=46)

| Class | Number | Serum creatinine (umol/L) | Proteinuria (g/24h) |
| --- | --- | --- | --- |
| I | 2014230 | 70.400 | 2.740 |
|  | 2011146 | 45.000 | 0.830 |
|  |  |  |  |
| IIa | 2013137 | 43.500 | 0.180 |
|  | 2014084 | 68.500 | 0.670 |
|  | 2011138 | 124.000 | 3.650 |
|  | 2012332 | 47.000 | 2.390 |
|  | 2015168 | 69.000 | 0.350 |
|  | 2015230 | 81.000 | 1.230 |
|  |  |  |  |
| IIb | 2013434 | 89.300 | 3.300 |
|  | 2013494 | 42.900 | 1.360 |
|  | 2014012 | 110.000 | 7.000 |
|  | 2014077 | 71.900 | 3.210 |
|  | 2014093 | 70.000 | 0.830 |
|  | 2014184 | 52.500 | 5.270 |
|  | 2014558 | 63.000 | 3.721 |
|  | 2014363 | 54.100 | 1.600 |
|  | 2014395 | 114.900 | 0.980 |
|  | 2015139 | 105.000 | 1.237 |
|  | 2012230 | 66.200 | 3.430 |
|  | 2011269 | 66.000 | 3.550 |
|  | 2011287 | 98.000 | 3.220 |
|  | 2011355 | 43.500 | 3.720 |
|  | 2011085 | 67.000 | 0.887 |
|  | 2015263 | 86.300 | 3.210 |
|  |  |  |  |
| III | 2013200 | 52.900 | 2.560 |
|  | 2013243 | 125.200 | 5.800 |
|  | 2013272 | 110.800 | 4.150 |
|  | 2013284 | 61.700 | 2.730 |
|  | 2013002 | 59.500 | 6.670 |
|  | 2013167 | 121.600 | 5.640 |
|  | 2014091 | 117.000 | 6.900 |
|  | 2014374 | 150.200 | 5.120 |
|  | 2014477 | 60.100 | 4.800 |
|  | 2014572 | 101.000 | 3.380 |
|  | 2014577 | 76.100 | 2.040 |
|  | 2015044 | 210.000 | 2.930 |
|  | 2015005 | 226.000 | 5.860 |
|  | 2015178 | 248.000 | 3.760 |
|  | 2015189 | 178.000 | 2.890 |
|  |  |  |  |
| IV | 2013573 | 164.000 | 6.060 |
|  | 2014424 | 189.500 | 2.700 |
|  | 2014569 | 277.800 | 9.420 |
|  | 2015034 | 197.000 | 3.090 |
|  | 2012187 | 260.000 | 3.280 |
|  | 2011308 | 227.000 | 5.600 |
|  | 2011092 | 127.000 | 2.790 |
