# Supplementary material for: Macrophage phenotype and its relationship with renal function in human diabetic nephropathy
Source: PLoS One. 2019 Sep 11;14(9):e0221991. doi: 10.1371/journal.pone.0221991 (PMC6738594; doi:10.1371/journal.pone.0221991)
Supplement: S2 Table — (RT-PCR). Control: 11.1mM glucose; HG: 25mM glucose; Results are the mean ± SD (n = 3–4 per group). aP<0.05 vs control. (DOC) [file pone.0221991.s003.doc]

Supporting Information

| S2 **Table.** The mRNA expression of iNOS, TREM-1 and MR of each group. (RT-PCR) | | | |
| --- | --- | --- | --- |
|  | Control | HG | Mannitol |
| iNOS | 0.914±0.079 | 4.119±0.053^a^ | 1.049±0.171 |
| TREM-1 | 1.124±0.060 | 9.876±0.117^a^ | 1.202±0.227 |
| MR | 4.362±1.280 | 1.832±0.572^a^ | 4.288±1.158 |

Control: 11.1mM glucose; HG: 25mM glucose; Results are the mean ± SD (n=3-4 per group). aP<0.05 vs control.
